# The EHA Research Roadmap: Hematopoietic Stem Cell Gene Therapy

**DOI:** 10.1097/HS9.0000000000000671

**Published:** 2022-02-04

**Authors:** Luigi Naldini, Maria Pia Cicalese, Maria Ester Bernardo, Bernhard Gentner, Michela Gabaldo, Giuliana Ferrari, Alessandro Aiuti

**Affiliations:** 1 San Raffaele Telethon Institute for Gene Therapy (SR-Tiget), IRCCS San Raffaele Scientific Institute, Milan, Italy; 2 Vita-Salute San Raffaele University, Milan, Italy; 3 Pediatric Immunohematology and Bone Marrow Transplantation Unit, IRCCS San Raffaele Scientific Institute, Milan, Italy; 4 Hematology and Bone Marrow Transplantation Unit, IRCCS San Raffaele Scientific Institute, Milan, Italy; 5 Fondazione Telethon, Milan, Italy

*In 2016, the European Hematology Association (EHA) published the EHA Roadmap for European Hematology Research*^1^ *aiming to highlight achievements in the diagnostics and treatment of blood disorders, and to better inform European policy makers and other stakeholders about the urgent clinical and scientific needs and priorities in the field of hematology. Each section was coordinated by 1 to 2 section editors who were leading international experts in the field. In the 5 years that have followed, advances in the field of hematology have been plentiful. As such, EHA is pleased to present an updated Research Roadmap, now including 11 sections, each of which will be published separately. The updated EHA Research Roadmap identifies the most urgent priorities in hematology research and clinical science, therefore supporting a more informed, focused, and ideally a more funded future for European hematology research. The 11 EHA Research Roadmap sections include Normal Hematopoiesis; Malignant Lymphoid Diseases; Malignant Myeloid Diseases; Anemias and Related Diseases; Platelet Disorders; Blood Coagulation and Hemostatic Disorders; Transfusion Medicine; Infections in Hematology; Hematopoietic Stem Cell Transplantation; CAR-T and Other Cell-based Immune Therapies; and Gene Therapy.*

## INTRODUCTION

**Figure 1.**
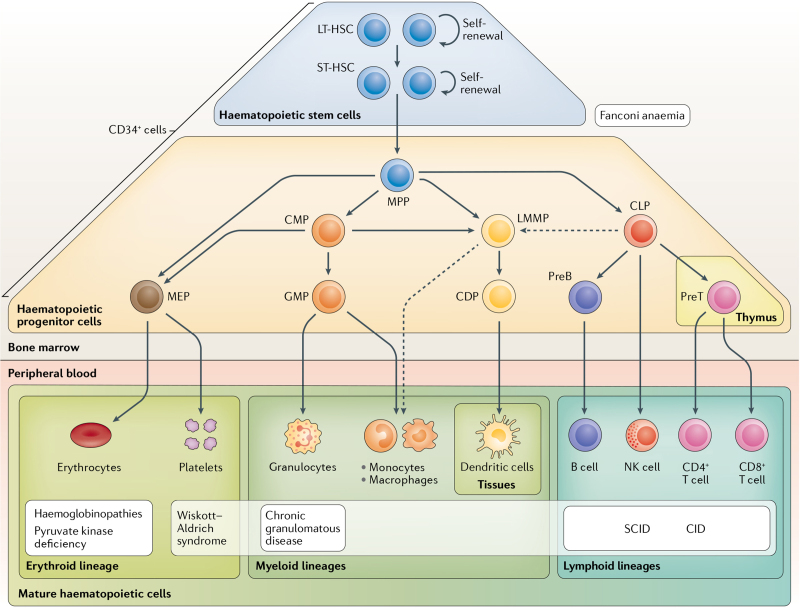
**Bone marrow-resident hematopoietic stem cells and hematopoietic progenitor cells replenish blood and tissues with new mature cells.^2^** Both hematopoietic stem cells and hematopoietic progenitor cells express the cell surface marker CD34, which is used to enrich a mixture of hematopoietic stem and progenitor cells for transplantation and gene therapy. Hematopoietic stem cells can be classed as long-term hematopoietic stem cells (LT-HSCs) or short-term hematopoietic stem cells (ST-HSCs). ST-HSCs progressively acquire lineage specifications to differentiate into lineage-committed progenitors and eventually terminally differentiated cells, which are released into the peripheral blood. A simplified scheme of human hematopoiesis is presented here. Alternative models have been postulated on the basis of cell surface marker analyses, in vitro and in vivo functional assays, clonal tracking by insertion analyses in hematopoietic stem and progenitor cell gene therapy studies, and single-cell RNA analyses (reviewed previously^22^). Mendelian genetic disorders can affect self-renewal, differentiation, and/or the function of different blood and immune cells. Examples of genetic diseases for which gene therapy is under investigation or approved are represented in white boxes below affected cell types. Wiskott–Aldrich syndrome affects platelets and other lineages. CDP = common dendritic progenitor; CID = combined immunodeficiency; CLP = common lymphoid progenitor; CMP = common myeloid progenitor; GMP = granulomonocytic progenitor; LMMP = lymphoid-myeloid primed progenitor; MEP = megakaryocytic–erythroid progenitor; MPP = multipotent progenitor; NK cell = natural killer cell; preB = pre-B cell; preT = pre-T cell; SCID = severe combined immunodeficiency. Reprinted with permission from *Nat Rev Genet*. 2021;22:216–234.

Genetic engineering of hematopoietic stem cells (HSCs) has progressed from early stage clinical trials providing evidence for substantial and durable benefits in some genetic deficiencies to the first Advanced Therapy Medicinal Products (ATMP) approved for the EU market (Figure 1).^2^ As safety and efficacy of HSC gene therapy (HSCGT) is further established by increasingly longer follow-up and a higher number of patients treated for different diseases, it may become a new pillar of treatment for several inherited monogenic affections for which allogeneic HSC transplantation (HSCT) represents a treatment option. Because HSCGT exploits autologous patient-derived cells, it has the following advantages over conventional allogeneic HSCT: (1) is in principle available to every patients; (2) does not entail the risk of graft-versus-host disease; (3) can lower substantially the risk of graft rejection, as the only potentially antigenic element in the administered cells is the therapeutic gene product; (4) can more easily establish partial chimerism with the administered HSC, which, according to disease and protocol, choice may allow lowering the requirement for myeloablative conditioning regimens and still provide substantial therapeutic benefit. Moreover, because of the genetic engineering step, gene correction may be designed to provide an even higher benefit than obtained with allogeneic normal or haploidentical carrier HSC, for instance by increasing therapeutic gene dosage in lysosomal storage diseases. HSCGT may eventually be exploited also for the treatment of some acquired affections, by instructing a new function to HSC or some of its progeny such as in gene-based delivery of a biotherapeutic or by establishing genetic resistance to an infectious agent.

On the other hand, HSCGT requires ex vivo culture and manipulation of the cells to enable genetic engineering, which in turn demands for establishing a suitable GMP-compliant multistep process from cells harvest to manufacturing and delivery at the clinical point-of-care. Furthermore, the culture as well as the genetic engineering procedure may affect some fundamental HSC biological properties negatively impacting the safety and efficiency of the procedure. Although this is an active area of investigation, most current protocols for growing HSC ex vivo fail to expand or even maintain for more than a few days the most primitive and long-term engrafting cells and both gene transfer and gene editing procedures have been reported to trigger innate cellular responses that may lead to delayed or halted growth, differentiation or even apoptosis and cell death. Consequently, if these adverse effects individually or cumulatively surpass a threshold during cell manufacturing, they may cause delayed hematopoietic recovery or even failure of engraftment, exposing the patient to high risk of infections, and also compromise the clonal composition of the engineered hematopoietic graft, which may reduce its long-term resilience and safety.

Last but not least, every meaningful genetic engineering of HSC requires stable, albeit local, modification of the cellular DNA sequence for ensuring life-long transmission to its cell progeny. This is a mutagenic procedure by definition, which entails a genotoxic risk dependent on the type (gene transfer versus editing), site, extent, and specificity of engineering. Whereas genotoxicity emerged as a significant risk in early HSCGT trials leading to leukemia development in some treated patients, it appears to be substantially alleviated by the new generation of lentiviral vectors currently in broader use. In any case, long-term monitoring of HSCGT patients is still required to establish the long-term safety and efficacy of these promising new therapies.

In the following sections, we highlight some key features of the genetic engineering platforms under clinical development for HSCGT, the current stage and future outlook of clinical applications of HSCGT in some major diseases families, and the regulatory implications of these novel and revolutionary medicines entering the clinical arena.

## GENE THERAPY PLATFORMS

### Introduction

Most current genetic engineering of HSC exploits 2 major strategies, gene replacement mediated by retroviral gene transfer vectors or targeted gene editing mediated by artificial sequence-specific endonucleases. Gene transfer strategies exploit a couple of well-established platforms that were developed in research laboratories between 15 and 20 years ago. Since then, progressive clinical testing in >300 patients worldwide for the treatment of several diseases have generated 2 ATMPs approved for the EU market with several more expected in the upcoming years (Table 1).^2^ Gene editing strategies are still in the earliest stage of clinical testing, although they are witnessing a remarkable and constant progress advancing the versatility, precision, and scope of the technological platforms adopted.^3,4^

### European research contributions

The EU has represented a privileged theatre for the clinical development of HSC GT based on retroviral gene transfer from the earliest pioneering clinical trials until today’s most advanced stages.

Retroviral vectors are replication-defective viral particles derived either from γ-retroviruses (γ-retroviral vectors [γRVs]), such as Moloney leukemia virus (MLV), or from the human lentivirus HIV-1 (lentiviral vectors [LVs]). They integrate semi-randomly a reverse transcribed RNA genome into the chromatin of the transduced cells. The vector genome comprises *cis*-acting signals for packaging, reverse transcription, and integration, lacks full length or open viral genes and comprises the therapeutic gene expression cassette. γRVs were the earliest to be developed, having limited efficiency of gene transfer into the more primitive HSC because productive infection is strictly dependent on concurrent replication of the targeted cell. Moreover, their early and most commonly used design exploited the strong enhancer/promoter sequences embedded in the long terminal repeats (LTRs) of the viral genome to drive transgene expression. This feature, coupled with an integration bias favoring insertion near the promoter of actively expressed genes, result in some likelihood of altering expression of endogenous genes nearby the insertion site through promoter insertion and enhancer-dependent trans-activation. Sporadic insertions near proto-oncogenes may result in activation of their oncogenic potential, thus endowing the transduced cell with a growth advantage leading to progressive clonal expansion in vivo and, in some cases, eventual transformation by further accumulation of mutations. Vector integration studies have allowed ascribing the origin of leukemia emerging in treated patients even several years post HSCGT to such types of genotoxic insertions, and longitudinal monitoring of the clonal composition of reconstituted hematopoiesis have shown the frequent expansion of clones carrying such insertions before any clinical signs of leukemia. Despite these hurdles, one of the earliest application of γRV HSCGT for the treatment of severe combined immunodeficiency caused by deficit of adenosine deaminase (ADA-SCID) has shown efficacy and better safety outcome than observed for other diseases and became the first HSCGT ATMP approved for the EU market in 2016.^5^

Because of the lower gene transfer efficiency and clinically relevant genotoxicity, the γRV platform has now been almost completely replaced by LV, which have improved both aspects.^1,6^ The key LV features underlying these improvements are: (1) exploitation of the HIV core capacity to infect nondividing cells to enhance transduction of the more primitive HSC after a short ex vivo stimulation; (2) advanced vector design which completely eliminates transcriptional activity from the vector LTRs. Expression of the therapeutic gene is driven from an internal promoter of choice, either reconstituted from the cellular promoter of the gene to be replaced, thus mimicking its physiological expression pattern, or from a moderately active house-keeping promoter, thus providing for ubiquitous expression. These features, combined with an insertion bias that favors the body rather than the promoter of expressed genes, strongly alleviate the risk of genotoxic effects at the insertion site; (3) exploitation of the HIV mechanism for packaging unspliced viral genome to transfer complex gene expression cassettes, such as used to establish erythroid specific and robust expression of a globin transgene in hemoglobinopathies. An ever-growing number of patients treated by LV HSCGT for several diseases have been showing stable and robust engineering of reconstituted hematopoiesis with highly polyclonal composition and without signs of genotoxicity such as expanding clones or enrichment for insertions at cancer gene. Depending on the disease, conditioning applied and product manufacturing, the engineered cell fraction can reach up to near completion, with evidence for distinct types of progenitors contributing to early recovery or steady-state output, which is mostly driven by engrafted self-renewing multipotent HSC.^2^

Gene editing strategies allow precise and targeted modification of a DNA sequence of choice, opening up novel and unique opportunities to genetic engineering. In the longest available and clinically more advanced version, it exploits an artificial endonuclease either composed of a sequence-specific DNA binding domain made of Zinc Finger or TALE protein modules coupled to a FokI nuclease half-domain (ZFN or TALEN), or of a target complementary single guide CRISPR RNA assembled with a Cas family nuclease (CRISPR/Cas).^4^ Either platform can deliver a DNA double-strand break (DSB) specifically in the target sequence, whose repair is exploited for the intended type of edit. Nontemplated DSB repair most commonly occurs by nonhomologous end joining (NHEJ), an error-prone process that often introduces small base insertion or deletion (indels) while sealing the break. If the break is targeted to a coding or an essential regulatory sequence the NHEJ outcome will most often be disruption or inactivation of the sequence.^7^ This represents the most frequent and, provided that the engineered nuclease is highly specific, better tolerated editing procedure. In most cases, electroporation is exploited to transiently introduce the nuclease mRNA or a preassembled CRISPR/Cas nucleoprotein into the cells to be edited. Transient but robust expression is required for efficient break induction while limiting toxicity. Nuclease specificity is crucial because DNA DSBs trigger a detrimental DNA damage response which, if robust and sustained, can lead to growth arrest, senescence, or apoptosis.^8^ Moreover, multiple DSBs increase the risk of genotoxicity and genomic rearrangements, such as translocations between different edited sites. Developing nuclease reagents with stringent specificity for the target site and comprehensively assessing off-target activity in preclinical models have been major goals of the field. A second more ambitious editing strategy exploits DNA DSB repair by homologous recombination (HR). This requires codelivery of the nuclease with a DNA template carrying the intended edit framed by homologous sequences to the DNA flanking the nuclease target site. If successful, this strategy will allow correcting mutations in situ, thus restoring both, function and physiological expression of the affected gene, and even inserting longer sequences, such as corrective cDNAs or transgene expression cassettes in safe genomic sites.^2^ The current challenges in applying HR-mediated editing to HSCGT is the relatively low efficiency of the process dependent on the poor permissiveness of primitive HSC to this pathway and the cumulative detrimental impact of inducing DNA DSBs and introducing an exogenous template DNA, which is best achieved by exploiting a viral vector such as adeno-associated vector.^9^

### Proposed research for the roadmap

While application of LV gene transfer can be envisaged to be tested in an increasingly larger number of diseases, the following areas of further HSCGT platform development can be proposed.

Because autologous HSCGT does not require full ablation and replacement of resident HSC, alleviation of the short-term and long-term morbidity of the procedure can be sought by careful targeting of chemotherapeutic regimens to the minimal required dose and through exploring emerging nongenotoxic conditioning strategies, such as those based on antibodies or immunotoxins targeting HSC.^10,11^ HSCGT may provide a favorable setting for early testing of these approaches, especially in diseases not requiring high levels of chimerism with functional cells for correction.

Further optimization of ex vivo HSC culture conditions, including more faithful reconstitution of signals supporting HSC self-renewal in bone marrow niches and/or their emergence from hemogenic endothelium in the embryo, testing novel combinations of transduction enhancers and uncovering their mechanism of action on HSC, and better standardization and more effective process control in ATMP manufacturing should allow improving the extent, predictability and reproducibility of gene transfer across different patients and treatments.

Close monitoring of clonal composition and HSC activity in the engineered hematopoietic graft of HSCGT patients will provide not only novel information on HSC biology in living humans but also help uncover clues to the long-term safety and robustness of therapeutic correction. As we learn more on the clinical relevance of clonal shrinking and skewing in aging hematopoiesis, it will be important to investigate whether ex vivo manipulation and genetic engineering may accelerate or aggravate such events. Delayed emergence of new HSC clones after HSCGT may highlight the capacity of engineered cells to return to long-term latency in vivo, establishing a safe reservoir for sustaining the graft in the case of emergencies or aging. On the other hand, if the growing number of treated patients and increasing time of follow-up were to lead to sporadic emergence of expanding dominant clones, we could learn about the residual extent and mechanism of genotoxicity of current vector design and introduce improved versions which are currently being investigated in stressed experimental models but await strong rational for clinical testing.

Gene editing strategies must be carefully monitored upon first clinical testing to uncover any adverse outcome of the procedure in terms of hematopoietic recovery and long-term engraftment, clonal composition of the engineered graft, and preserved long-term maintenance and multipotency of the edited HSC. The occurrence of large-scale genomic alterations in some edited cells and their in vivo fate will be investigated with the goal to decrease their occurrence or purge them from the product, if necessary. Protocols allowing for improved efficiency and tolerability of editing, especially when exploiting HR, will be developed for instance by adopting emerging ex vivo HSC expansion strategies and refined editors, or inhibiting or counteracting the detrimental responses induced by the procedure.

New gene editing platforms that bypass the requirement for DNA DSB, such as base editors and prime editors will be tested in preclinical studies for potential application to HSCGT investigating their efficiency, tolerability, and specificity, potentially providing for more precise and uneventful genetic engineering.^4^

The potential adverse effects of pre-existing immunity to gene transfer and, in particular gene editing reagents, some of which are of bacterial origin, will be investigated. Residual antigenic components might be present in the cell product and trigger cellular responses leading to clearance of the administered cells. The impact of pre-existing effector or regulatory T cells specific for the viral/editor component at different levels in the recipient will be investigated.

### Anticipated impact of the research

We are at the beginning of a new era of medicine exploiting genes and cells as novel therapeutics, leveraging on powerful genetic engineering tools to modify cell function and capture fundamental biological processes, such as HSC-driven reconstitution of intra and extravascular hematopoietic populations for therapeutic purposes. These approaches open unprecedented opportunities and largely unexplored paths for therapeutic interventions. Remarkable and durable benefits in until now orphan and otherwise severe and lethal diseases have been reported and are expected to be further achieved in growing numbers. However, this promise comes also with a tremendous challenge to the currently established framework for developing, regulating and distributing medicines while maintaining their economic sustainability by public and private healthcare providers and guaranteeing fair access to the patients. To address this challenge and attain the benefits that may come from this research, we must continue to support research establishments that produce innovation, facilitate the creation of start-ups promoting early development of the new technologies, advance education of biomedical trainees to become familiar with the new technologies and treatments, update regulatory requirements to emerging platforms and a rapidly evolving landscape, and foster engagement of pharmaceutical industries in clinical development and commercial deployment of the new ATMPs.

## GENE THERAPY FOR PRIMARY IMMUNODEFICIENCIES

### Introduction

Primary immune deficiencies (PIDs) are a heterogeneous group of >400 genetic diseases characterized by poor or absent function of one or more components of the immune system, whose main clinical manifestations comprise increased frequency and severity of infection, autoimmunity, and aberrant inflammation and malignancy.^12^ Early diagnosis and treatment remain a mainstay for all forms of PIDs to prevent organ damage and life-threatening infections and to improve prognosis and quality of life. Allogeneic HSCT is curative for many PIDs, but still carries a risk of mortality and morbidity from rejection, toxicity, and graft-versus-host disease. SCIDs were the first monogenic disorders for which HSCGT has been successfully developed. Clinical trials with promising results are ongoing for at least 6 different PIDs due to genetic defects of adaptive and/or innate immunity while a growing number of diseases are at preclinical stage of development.^2,13^

### European research contributions

European academic research played a key role in the development of successful HSCGT approaches for PID. In the past 2 decades, the European Commission funded collaborative research networks such as CONSERT, PERSIST, Clinigene, CELL-PID, NET4CGD, SCIDNET, SUPERSIST, and UPGRADE. Over 100 PID patients have been treated in early trials of HSCGT with γRV, showing reconstitution of immunity in most patients affected by SCID-X1 due to IL2RG deficiency, ADA-deficient SCID, Wiskott–Aldrich Syndrome (WAS), and X-linked Chronic Granulomatous Disease (X-CGD).^2^ ADA-SCID GT was the first PID for which non myeloablative conditioning was introduced and in 2016 became the first HSCGT approved in the EU (Strimvelis, Orchard Therapeutics Ltd, London, UK), with the indication of standard treatment for ADA-SCID lacking a compatible family donor^5^ Infusion of ADA gene replaced HSC resulted in persistent (>15 y) engraftment of gene-marked cells ranging from 1% to 10% in the myeloid compartment and reaching up to 100% in the lymphoid compartment, correction of adenosine metabolism and improved T-cell counts, leading to discontinuation of prophylaxis and decreased incidence of severe infections.^14^ The promising clinical data obtained in γRV trials have been tempered by the later development of T-lymphoblastic leukemia and myelodysplasia as a result of insertional mutagenesis. The incidence varied among disease types, suggesting that disease background, transgene function, and individual genetic predisposition influence tumorigenicity.

LV-based platform has been deployed in the past decade enabling more effective and safe insertion of therapeutic genes into HSC, with no evidence of leukemogenesis reported to this date. LV-HSCGT clinical trials for SCID-X1 and ADA-SCID have shown improved lymphocyte counts with clinical benefit.^15^ HSCGT for WAS resulted in amelioration of immune functions, including autoimmunity, and reduced incidence of severe bleeding events.^3,16^ Moreover, HSCGT-mediated restoration of oxidase function in X-CGD patients leading to protection from bacterial and fungal infections in most treated patients.^17^

### Proposed research for the roadmap

Continuous patients monitoring, also through analyses of vector insertion sites, will be required to confirm long-term safety and efficacy of patients who underwent HSCGT. Clinical trials for artemis deficiency, LAD-1, and osteopetrosis have recently started.^2^ Preclinical studies are actively pursued for other PIDs in which HSCGT could represent an alternative to allogeneic HSCT, also in the most severe or adult patients presenting with ongoing infections and/or organ damage including JAK3-SCID, PNP deficiency, RAG1/2 deficiency, ZAP70 deficiency, Munc 13-4 deficiency, and DADA2.^18^ HSCGT could also become available for less severe phenotypes, in which alloHSCT would not be indicated such as BTK deficiency. New gene editing technologies have the potential to circumvent some of the problems associated with viral gene addition and could be suitable for diseases requiring physiological regulation of gene expression or inactivation of dominant alleles. Preclinical proof of concept has been attained in correcting gene mutations for IL2RG, WAS, p47-CGD, CD40L deficiency, and IPEX, and clinical testing of these strategies is awaited.

One of the key factors in the success of HSCGT for SCID and WAS relies on the selective advantage of functionally corrected lymphoid cells, previously observed in patients with somatic revertants and allogeneic HSCT. Thus, even in patients not receiving conditioning, active thymopoiesis was shown to be maintained for many years after treatment, suggesting durable thymic engraftment of long-lived lymphoid progenitors.^2^ On the other hand, conditioning is required to achieve polyclonal engraftment of gene-corrected HSC, restore normal B-cell lymphopoiesis and establish corrected myelopoiesis. SCIDs seem ideal candidates to explore the use of conditioning mediated by monoclonal antibodies or immunotoxins for HSCGT while exploiting the selective advantage in the lymphoid lineage. Preliminary results of a clinical trial in the context of allogeneic HSCT for SCID-X1 show sufficient degree of HSC engraftment.^11^

GT with autologous mature lymphocytes or lymphoid progenitors has also been explored preclinically with the rationale to provide in patients not eligible to allogeneic HSCT or HSCGT, immune responses to infection in diseases such as CD40L or perforin deficiency as well as control of immune dysregulation regulation in FOXP3 deficiency.

### Anticipated impact of the research

Gene therapy for PIDs is moving from being an experimental approach to approved drug products that are routinely beneficial. The expansion of gene addition strategies and implementation of gene editing approaches, together with the standardization of the technology, will be important for increasing the armamentarium of approved therapies as an alternative to allogeneic HSCT.

## HEMOGLOBINOPATHIES

### Introduction

Hemoglobinopathies are genetic defects of hemoglobin chain production, caused by mutations in the α- or β-globin gene clusters. The most frequent and severe are transfusion-dependent β-thalassemia (TDT) and sickle cell disease (SCD), characterized by a reduced or absent level of adult hemoglobin (HbA) and the production of an abnormal structural variant (HbS), respectively. In TDT, survival of patients depends on chronic blood transfusion associated to iron chelation. In SCD, sickling of deoxygenated red blood cells (RBCs) promotes painful vaso-occlusive crises, acute chest syndrome, stroke, and eventually death. Blood transfusion and fetal hemoglobin (HbF) induction by hydroxyurea represent current treatments. Allogeneic HSCT is curative for both diseases, but with limited availability of suitable donors and variability in clinical outcome depending on patient’s age. LV-mediated HSCGT relies on the erythroid specific expression of a normal gene copy and has been recently approved in Europe for patients over 12-years-old affected by less severe β-thalassemia mutations, and it is still in experimental trials for SCD.^2^ Gene editing strategies in the field of hemoglobinopathies have reached the stage of clinical testing, with ongoing phase 1/2 trials using ZFN or CRISPR/Cas technologies in SCD and in TDT. Reactivation of HbF synthesis by disruption of Bcl11a suppressor led to initial encouraging clinical results^19^ and longer follow-up will fully disclose the real potentiality as well the caveats of this novel approach.^20^

### European research contributions

European researchers have been prime actors in nonclinical and clinical research for the cure of TDT and SCD, contributing to the discovery of the disease molecular cause and the molecular mechanisms of globin genes regulation, introducing allogeneic HSCT and translating basic research to clinical application of gene therapy. Two successful clinical trials in TDT have been conducted in France^21^ and Italy,^22^ paving the way for application also in SCD.^23^ The results showed correction of the disease in most adult patients carrying nonsevere mutations and in young patients with severe mutations. The level of marked engrafted cells positively correlates with the clinical benefit, highlighting a threshold of genetically corrected cells to produce sufficient HbA *per* cell to rescue anemia and ineffective erythropoiesis in TDT, and to dilute abnormal HbS and preventing sickling in SCD. The functional status of HSC and the BM microenvironment is particularly relevant in TDT, where an impaired HSC-niche cross-talk has a negative impact on HSC functionality.^24^

### Proposed research for the roadmap

Gene therapy for hemoglobinopathies poses unique challenges, including high level of transgene expression for therapeutic correction and high global incidence worldwide. A single ATMP on the market (Zynteglo) will not be sufficient for such a prevalent disease but demonstration of dissimilarity of drug products might be challenging, thus discouraging biotech and/or pharma investments in further academic preclinical and clinical research. New rules governing the ATMP market should be introduced to favor the treatment of large patients’ population with biologically similar products. As far as for other genetic diseases, the myeloablative conditioning required to favor the engraftment of genetically modified HSPCs imposes a high burden on patients, with general toxicity and impairment of fertility. Non-genotoxic biological conditioning specifically targeting resident HSC would be highly desirable, although its efficiency needs to be tested and modeled in the context of a BM engulfed by an expanded erythroid component. Additionally, amelioration of BM microenvironment will be instrumental in favoring engraftment and expansion of HSCs in the context of both, allogeneic HSCT and HSCGT.

### Anticipated impact of the research

The long-term effort in GT research for TDT has recently resulted in the approval of a first ATMP (Zynteglo) and poses the challenge of its manufacturing and distribution to a large number of affected patients. The development and optimization of gene editing approaches will offer an additional opportunity of intervention, with potentially therapeutic levels of Hb production exploiting the power of chromosomal regulatory sequences. In both cases, the complex manufacturing and associated costs represent major obstacles for fulfilling the current medical need, leaving the door open to the development of novel and potentially more feasible approaches of in vivo gene therapy.

## METABOLIC DISORDERS

### Introduction

Inborn errors of metabolism (IEMs) are a large class of genetic disorders characterized by the absence/dysfunction of an enzyme or its co-factor leading to disruption of cellular biochemical functions. Subgroups characterized by permanent, progressive symptoms often involving the nervous system and the skeleton encompass peroxisomal and lysosomal storage diseases (LSDs), according to the subcellular location of the defects. LSD pathology results from accumulation of waste materials, causing cellular dysfunction, alteration of cell morphology, impaired autophagy, oxidative stress, neuroinflammation, and impaired organ function in the brain, bones, viscera, and connective tissue.^25^ Enzyme replacement therapy (ERT) and allogeneic HSCT (allo-HSCT) have been exploited as potential treatments for LSDs, but preclinical and clinical studies have shown limited efficacy in ameliorating cardinal disease manifestations. GT using autologous HSC is an attractive alternative to allo-HSCT, as it promises not only an improved safety profile, but also enhanced efficacy, due to its potential to turn HSC progeny into hyperfunctional enzyme factories capable of more effectively cross-correcting nonhematopoietic cells, systemically and locally within the affected tissues.

### European research contributions

X-ALD (X-linked adrenoleukodystrophy): Successful treatment of 2 boys affected by the childhood cerebral form of adrenoleukodystrophy (CALD) with LV-transduced HSC marked a milestone ushering in a new era of more effective and less genotoxic engineering of hematopoiesis.^26^ Since then, >30 patients have been treated with HSCGT,^27,28^ which arrested disease progression and kept children treated early in their disease course free from major functional disabilities, similar to what can be achieved by a successful Allo-HSCT.

MLD: Metachromatic leukodystrophy put LV HSCGT to a stress test, as allo-HSCT is not effective in early onset forms of the disease and, based on preclinical models, benefit could only be expected from supraphysiologic expression of ARSA enzyme from the progeny of transduced HSC. A clinical trial of HSCGT in presymptomatic or early symptomatic early onset MLD^29^ showed unprecedented levels of stable gene marking throughout hematopoiesis, effective prevention of disease onset in presymptomatic patients and slowing of disease progression in early symptomatic ones, thereby representing the first treatment capable of modifying the natural history of this devastating neurologic disease. This MLD HSCGT has recently been granted market authorization by EMA (commercial name “Libmeldy”).

MPSIH (mucopolysaccharidosis type 1-Hurler syndrome): Capitalizing on the increased therapeutic potential of supraphysiologic enzyme reconstitution by HSCGT in MLD, a phase I/II study for Hurler disease, the most severe form of MPSIH, has recently been conducted, introducing a shortened ex vivo manipulation protocol with PGE2 as transduction enhancer that allowed optimal conservation of repopulation potential. Rapid biochemical correction and supraphysiologic IDUA activity was obtained in all 8 treated patients, with an excellent safety profile.

MPSIIIA (mucopolysaccharidosis type IIIA): Similarly encouraging preliminary results have been obtained in a phase I/II trial for MPSIIIA.^30^ HSCGT allowed achieving supraphysiologic enzyme levels in hematopoietic lineages and effective substrate reduction, consolidating the potential of gene therapy in this group of LSDs.

Investigational HSCGT is currently being studied in clinical trials in Fabry disease, Gaucher disease, and cystinosis in the United States and Canada.

### Proposed research for the roadmap

HSCGT has been demonstrated to represent a promising treatment modality for previously refractory LSDs and other metabolic disorders. Ongoing and future clinical trials will provide essential insights into the mechanisms of correction through HSCGT in specific tissues/organs (ie, nervous system, skeleton) and will allow refinement of indications and procedures of treatment (ie, conditioning regimens, cell dose, modulation of antitransgene immune response and, potentially, intrathecal administration of corrected HSPC). LSDs, and in particular MPSs, represent an area of further HSCGT development with more diseases potentially amenable to cross-correction mechanisms and benefitting from supraphysiologic levels of the missing enzyme.

HSCGT-based therapeutic strategies have provided the clinical basis for the implementation of newborn screening (NBS) for IEMs which allows early diagnosis and timely treatment, this being a key factor for better preserving tissue and organ function and improving treatment outcome. NBS programs for LSDs are being conducted in Europe and the United States to this purpose.

### Anticipated impact of the research

The last 10 years represent unprecedented times for HSCGT in IEMs with several clinical approaches being developed and tested in Phase I/II clinical trials. So far, HSCGT in LSDs has provided evidence of metabolic correction in several diseases (MLD, MPSIH, and MPSIIIA), while demonstration of robust and sustained clinical efficacy has been obtained so far in ALD and MLD. Longer observation of HSCGT-treated IEM patients will give support to the clinical efficacy of these strategies in the long-term, together with additional proof of safety. Further clinical development of HSCGT approaches in these and other IEMs will possibly transform the outcome of some of them.

## OTHER EMERGING APPLICATIONS

A long-sought application of HSCGT has been the correction of Fanconi anemia (FA), caused by loss-of-function mutations in any one of at least 17 genes in the FA pathway and resulting in the inability to repair interstrand DNA crosslinks. HSCs are most sensitive to this defect and progressively exhaust, giving rise to severe pancytopenia as well as a high risk of myelodysplasia. Whereas GT has been particularly challenging for this disease because of the paucity and fragility of FA HSC, the growth advantage of corrected HSC provides the opportunity for in vivo expansion of even a limited engrafted input. A recent trial of LV–based HSC GT conducted in Spain for FA complementation group A reported successful engraftment of corrected HSC in the absence of conditioning followed by sustained and progressive expansion of the corrected hematopoiesis, reaching therapeutic relevance over several months.^31^ Preclinical studies of HSC gene editing are also ongoing in FA, aging leveraging on the selected advantage of correction.

Another recent development of HSC GT is to broaden gene addition beyond the replacement of a defective gene and encompassing the delivery of a biotherapeutic to disease sites or establishing resistance to an ineradicable infectious agent. These approaches are based on engineering hematopoietic progenitors with vectors designed to selectively express in certain hematopoietic lineages, differentiation or maturation stages. For gene-based delivery of biotherapeutics, the engineered mature cells act as smart agents selectively distributing the gene product to extravascular disease sites. Emerging application of this approach are being tested to target immunostimulatory cytokines, such as alpha-interferon, to tumors by tumor-infiltrating macrophages or immunomodulatory/growth-promoting agents to neuroinflammatory/degenerative lesions by brain-infiltrating macrophages. Overall, these early stage studies may open up new avenues for the treatment of some prevalent affections, such as cancer, chronic infections and neurodegenerative diseases.

## REGULATORY CHALLENGES

HSCGT ATMPs have unique attributes which differentiate them from standard pharmaceuticals and biologics. They bring the potential to offer a durable curative therapeutic effect upon a single administration to patients who may have few or no alternative treatment options. However, the complexity and novelty of these ATMPs also present several challenges that need to be overcome to ensure that these products reach all those in need.

Regulators have established dedicated pathways and expert committees to ensure appropriate and expedited registration of ATMPs. In EU, specific regulatory tools are available, such as *classification* of the products as ATMP and *certification* of ATMP quality and nonclinical data for small and medium-sized enterprises (SMEs). In addition, the use of more general tools such as *scientific advice/protocol assistance*, *parallel EU/US scientific advice*, the priority medicines or *PRIME scheme* and *qualification of novel methodologies,* is recommended to discuss as early as possible the strategy under development with the central (ie, EMA in EU or FDA in the United States) and national regulatory authorities that will assess the investigational drug application. However, it should be recognized that these novel and revolutionary medicines entering the clinical arena pose great challenges for the developers as there is still lack of harmonization of regulatory requirements across geographic areas in terms of quality standards, genetic-modified organisms (GMOs) manipulation, preclinical data packages to support the first in human and clinical evidence to be generated for registration and access to the market. Moreover, most products are developed to treat rare and orphan diseases with the associated challenges of small patient population and complex clinical trial design. HSCGTs are innovative products which bring complexity in the manufacturing process and in the comprehensive analytical panel needed to characterize, control and release the product, which require highly specialized manufacturing equipment, processes and skills. Ensuring consistent standards and adequate characterization across starting materials, processes and infrastructure is a common challenge for HSCGT manufacturing. As HSCGT are highly personalized medicines they pose even higher challenges in terms of standardization and reproducibility. Overall, these hurdles together with the associated high costs and regulatory burden may jeopardize early steps toward clinical testing of new GT products, especially if developed within the context of academic institutions. Thus, a critical balance should be maintained when manufacturing products for early stage clinical testing by adopting quality standards sufficient to ensure patient safety as well as compliance with regulatory requirements for clinical testing while postponing more stringent requirements and comparability studies to later stages of clinical development. At the same time, it should be realized that entering the clinic with a nonoptimized product may compromise its effective development and, even if shown to be safe and efficacious, may delay the registration due to the further time needed for process scale-up and full validation for commercialization.

Despite different regulatory requirements across regions still exist for this kind of therapies, there is an emerging paradox between regulators’ approaches implemented to ensure rapid approval and early access to the ATMP, and payers’ and health technology assessment (HTA) bodies’ current hesitancy to ensure access until the long-term safety and efficacy profile has been fully characterized. Payers/HTA bodies need to establish more effective mechanisms to capture the full benefits of these therapies and overcome current potential barriers to timely patient access postapproval. Early dialogue with HTA bodies and future payers during ATMP development using tools like the *parallel consultation with HTAs* might help to address the challenges posed by these transformative medicines and develop new evaluation tools and payment models to support financial sustainability for both payers and manufacturers and make HSCGT fairly and promptly available to patients.

**Table 1.** Most Recent HSPC Gene Therapy Available on clinicaltrails.gov (Recruiting or Completed)

Disease *(Gene*)	Trial phase	Vector	Conditioning	Preliminary Outcomes	Clinical Trial Registry Number (Reference)
SCID-X1 (*IL2RG*)	I/II	G2SCID LV	Low-dose busulfan	Still recruiting	NCT03311503
SCID-X1 (*IL2RG*)	I/II	G2SCID LV	Low-dose busulfan	Still recruiting	NCT03601286
SCID-X1 (*IL2RG*)	I/II	TYF-IL-2Rg self-inactivating LV (TYF-IL-2Rg)	Not known	Still recruiting	NCT03217617
SCID-X1 (*IL2RG*)	I/II	LV VSV-G pseudotyped CL20- 4i-EF1a-hyc-OPT	Low-dose busulfan	Sustained marking levels and restoration of humoral responses to immunization	NCT01306019 (De Ravin, *Sci Transl Med*. 2016;8:335ra57)
ADA-SCID (*ADA*)	I/II	EFS-ADA LV	Low-dose busulfan	Sustained engraftment of genetically modified HSPCs in all the patients with long-term metabolic detoxification from deoxyadenosine nucleotides after stopping ERT	NCT02999984 (Kohn, *Blood* (2019) 134 (Supplement_1): 3345)
ADA-SCID (*ADA*)	I/II	EFS-ADA LV	Low-dose busulfan	Sustained engraftment of genetically modified HSPCs in 9/10 patients	NCT01852071 (Kohn, Blood (2019) 134 (Supplement_1): 3345)
ADA-SCID (*ADA*)	I/II	EFS-ADA LV	Low-dose busulfan	Still recruiting	NCT03765632
ADA-SCID (*ADA*)	II/III	EFS-ADA LV	Low-dose busulfan	Suspended (recruitment on hold for business reasons)	NCT04140539
ADA-SCID (*ADA*)	Not Applicable^a^	Self-inactivating LV TYF-ADA	Not known	Still recruiting	NCT03645460
WAS (*WAS*)	I/II	w1.6_hWASP_WPRE (VSVg) LV	Reduced-intensity conditioning regimen with busulfan and fludarabine	Sustained engraftment of genetically modified HSPCs with reduction of bleeding events and restoration of WASP expression in lymphocytes and plateles,	NCT01515462 (Ferrua et al^16^)
WAS (*WAS*)	I/II	w1.6_hWASP_WPRE (VSVg) LV^b^	Myeloablative conditioning regimen with busulfan and fludarabine	Sustained multi-lineage vector gene marking over time. All subjects had improvement or resolution of eczema and none had intercurrent severe infectious events	NCT01410825 (Labrosse, *Blood* (2019) 134 (Supplement_1): 4629)
WAS (*WAS*)	II	w1.6_hWASP_WPRE (VSVg) LV^b^	Reduced-intensity conditioning regimen of busulfan and fludarabine	No results available	NCT03837483
WAS (*WAS*)	I/II	w1.6_hWASP_WPRE (VSVg) LV^b^	Myeloablative conditioning regimen with busulfan and fludarabine	Stable engraftment of genetically and functionally corrected lymphoid and myeloid cells in all patients with lack of severe adverse events or clonal expansion	NCT02333760 (Magnani, et al. *Mol Therapy* 2020;28:4S1)
X-CGD (*gp91phox*)	I/II	G1XCGD LV	Myeloablative conditioning regimen with busulfan	Stable vector copy numbers and persistence of oxidase-positive neutrophils in 6/7 surviving patients. No new CGD-related infections	NCT01855685 and NCT02234934 (Kohn et al^17^)
X-CGD (*gp91phox*)	I/II	G1XCGD LV	Myeloablative conditioning regimen with busulfan	2/4 patients showed clinical and biological benefits	NCT02757911 (Magnani, et al. *Mol Therapy* 2020;28:4S1)
Transfusion-dependent β-thalassemia (*HBB*)	I/II	GLOBE LV	Myeloablative conditioning with treosulfan and thiotepa	Robust and persistent engraftment of genetically modified HSPCs in 7/9 patients and achievement of transfusion independence in 4/6 children	NCT02453477 (Scaramuzza, et al. *Mol Therapy* 2020;28:4S1)
Transfusion-dependent β-thalassemia (*HBB*)	III	LV βA-T87Q-Globin	Myeloablative conditioning regimen with busulfan	Transfusion independence was observed in most of the patients. HbAT87Q stabilized approximately 6 months after treatment and patients who stopped RBC transfusions had improved erythropoiesis	NCT02906202 (Thompson, et al. *Blood,* 2019; 134(Supplement_1): 3543)
Transfusion-dependent β-thalassemia (*HBB*)	III	LV βA-T87Q-Globin	Myeloablative conditioning regimen with busulfan	In 3/4 patients with ≥ 6 months follow-up have stopped transfusions and one patient has achieved transfusion independence	NCT03207009 (Lal, *Blood,* 2019, 134(Supplement_1): 815)
SCD (*HBB*)	I/II	LV βA-T87Q-Globin	Myeloablative conditioning with busulfan	Improvement in hematologic parameters and disease-related symptoms	NCT02151526 (Magrin, *Blood,* 2019, 134 (Supplement_1): 3358)
SCD (*HBB*)	I/II	LV βA-T87Q-Globin	Myeloablative conditioning with busulfan	Reduction in the annualized rate of disease-related symptoms. Patients maintained HbAT87Q production, demonstrating the durability of gene therapy-derived β-globin gene expression	NCT02140554 (Walters, *Blood,* 2019, 134 (Supplement_1): 2061)
SCD (*HBB*)	I/II	Gamma-globin LV	Reduced intensity conditioning with melphalan	Still recruiting	NCT02186418
SCD (*HBB*)	I/II	GLOBE1 LV expressing the βAS3 globin gene	Myeloablative conditioning with busulfan	Still recruiting	NCT03964792
SCD (*HBB*)	I/II	Lenti/G-βAS3-FB LV	Myeloablative conditioning with busulfan	Still recruiting	NCT02247843
SCD (*HBB*)	I	LV encoding human γ-globinG16D and short-hairpin RNA734 for selection of hypoxanthine guanine phosphoribosyltransferase	Reduced intensity conditioning with melphalan	Still recruiting	NCT04091737
MLD *(ARSA*)	I/II	LV ARSA	Myeloablative conditioning with busulfan	Sustained multilineage engraftment of genetically modified HSPCs and clinical improvement compared to natural history patients	NCT01560182 (Fumagalli WORLD symposium 2020)
MLD *(ARSA*)	II	LV ARSA	Myeloablative conditioning with busulfan	Preliminary results showed multilineage engraftment of genetically modified HSPCs and restoration of ARSA activity	NCT03392987 (Fumagalli WORLD symposium 2020)
X-ALD *(ABCD1*)	II/III	SIN LV MNDprom-ABCD1 (Lenti-D) encoding human adrenoleukodystrophy protein	Myeloablative conditioning with busulfan and cyclophosphamide	Gene marked cells after engraftment and measurable ALD protein in all the patients	NCT01896102 (Eichler et al^27^)
X-ALD *(ABCD1*)	III	Autologous CD34+ cells transduced with SIN LV MNDprom-ABCD1 (Lenti-D) encoding human adrenoleukodystrophy protein	Myeloablative conditioning with busulfan and fludarabine	Still recruiting	NCT03852498
MPSI *(IDUA*)	I/II	LV IDUA	Myeloablative conditioning with busulfan and fludarabine	Preliminary results showed multilineage engraftment of genetically modified HSPCs and restoration of IDUA activity	NCT03488394 (Gentner, WORLD symposium 2020)
MPSIII *(SGSH*)	I/II	CD11b LV vector encoding for human SGSH	Myeloablative conditioning with busulfan	Preliminary results showed multilineage engraftment of gene-modified cells and sustained vector copy number	NCT04201405 (Kinsella et al. *Mol Therapy* 2020;28)
Fabry disease (*GLA*)	I	LV AVR-RD-01	Myeloablative conditioning	Durable engraftment. Sustained plasma and leukocyte enzyme activity	NCT02800070 (AvroBio data update, ASGCT 2020)
Fabry disease (*GLA*)	I/II	LV AVR-RD-01	Myeloablative conditioning	Durable engraftment. Sustained plasma and leukocyte enzyme activity	NCT03454893 (AvroBio data update, ASGCT 2020)

^a^Not applicable is used to describe trials without FDA-defined phases.

^b^The same vector design but performed in different manufacturing sites and with different transduction protocols.

Adapted from: Tucci F, Scaramuzza S, Aiuti A, Mortellaro A. Update on clinical ex vivo hematopoietic stem cell gene therapy for inherited monogenic diseases. *Mol Ther*. 2021;29:489-504.

**Summary box: Main research & policy priorities**

1. Expand administration of HSCGT to increasing number of patients and continue long-term monitoring of the treated ones to establish safety and efficacy of the treatment and make it first-line option for those diseases in which an autologous source of gene-corrected cells can lower the risks and improve the benefits of allogeneic HSCT.
2. Broaden application of HSCGT to disease families where strong proof-of-principle of the therapeutic potential of HSCGT has been demonstrated, such as LSD.
3. Testing the therapeutic potential of emerging nongenotoxic conditioning regimens for which HSCGT may provide a favorable setting.
4. Improving the extent, predictability, and reproducibility of lentiviral gene transfer across different patients and treatments.
5. Monitoring of clonal composition and HSC activity and fate in the long-term genetically engineered graft.
6. Investigate the multifactorial contribution and longitudinal stepwise evolution of genotoxicity in HSCGT and devise strategies to further alleviate the risk of progression to malignancy.
7. Closely monitor new gene editing strategies as they enter clinical testing to uncover any adverse outcome of the procedure in terms of hematopoietic recovery and long-term engraftment, clonal composition of the engineered graft, and preserved long-term maintenance and multipotency of the edited HSC.
8. Investigate and ameliorate any disease-specific alterations of the BM microenvironment to facilitate engraftment and expansion of gene corrected HSC.
9. Assess the potential adverse effect of pre-existing or newly developed immunity to gene transfer and editing tools as well as to transgene products as HSCGT is applied to an increasing number of diseases and lymphodepleting preconditioning is further alleviated or bypassed.
10. Establish more supportive framework for developing, regulating and distributing ATMP while maintaining their economic sustainability by public and private healthcare providers and guaranteeing fair access to the patients, also by adapting quality standards to the stage of development and allowing development of biologically similar products.

## DISCLOSURES

MPC is PI or Co-PI of studies sponsored by Orchard Therapeutics, and consulting for Orchard Therapeutics, Exafield S.r.l, LSC Italy S.r.l, Atheneum S.r.l. MEB is PI of a clinical trial sponsored by Orchard Therapeutics and participated in an Advisory Board for Orchard Therapeutic. BG is a founder, stockholder and consultant of Genenta Science. MG is consulting for Genespire. AA is the PI or Co-PI of clinical trials sponsored by Orchard Therapeutics. LN is a founder, owns equity, and is consultant and member of the scientific advisory board of Genenta Science, Genespire, Epsilen Bio/Chroma, Tessera Therapeutics, Magenta Therapeutics. All the other authors have no conflicts of interest to disclose.
